# Surveillance for Invasive *Salmonella* Disease in Bamako, Mali, From 2002 to 2018

**DOI:** 10.1093/cid/ciaa482

**Published:** 2020-07-09

**Authors:** William L Still, Milagritos D Tapia, Sharon M Tennant, Mamadou Sylla, Aliou Touré, Henry Badji, Adama Mamby Keita, Samba O Sow, Myron M Levine, Karen L Kotloff

**Affiliations:** 1 Department of Epidemiology and Public Health, University of Maryland Graduate School, Baltimore, Maryland, USA; 2 Department of Pediatrics and Center for Vaccine Development and Global Health, University of Maryland School of Medicine, Baltimore, Maryland, USA; 3 Centre Pour le Développement des Vaccins–Mali, Bamako, Mali

**Keywords:** nontyphoidal *Salmonella*, typhoid fever, surveillance, Mali, invasive bacterial infections

## Abstract

**Background:**

*Salmonella enterica* bloodstream infections are an important cause of childhood morbidity and mortality, including in Mali. We report 17 years of surveillance for nontyphoidal and typhoidal *S. enterica* infections among inpatients and outpatients at l’Hôpital Gabriel Touré, the main source of pediatric tertiary care in Bamako, Mali.

**Methods:**

Between June 2002 and December 2018, a blood culture was collected from 54 748 children aged ≤15 years with fever and/or suspected invasive bacterial infection who provided consent (38 152 inpatients, 16 596 outpatients). Bacterial pathogens were identified using standard microbiological techniques and serovars of *S. enterica* were determined by PCR and/or agglutination with antisera.

**Results:**

Nontyphoidal *Salmonella* (NTS) was identified in 671 enrolled inpatients (1.8% of all enrolled inpatients, 13.8% of enrolled inpatients with a positive culture). *S*. Enteritidis, the most common NTS serovar, accounted for 38.5% of all NTS isolates (n = 258), followed by *S.* Typhimurium (31.7%, n = 213). The median (SD) age of children with a culture positive for NTS was 1.8 (3) years. Overall case fatality was 20.9%. An additional 138 inpatients (0.4%) had a positive culture for typhoidal *Salmonella*. NTS was identified in 11 outpatients (0.07%), while typhoidal *Salmonella* was found in 49 outpatients (0.3%). The annual incidence of invasive NTS disease decreased over the study period, but case fatality remained high.

**Conclusions:**

Although incidence decreased, NTS remained a major cause of invasive bacterial infection and mortality among hospitalized children in Bamako, while typhoidal *Salmonella* was uncommon. Because 87% of NTS belonged to only 4 serovars, a multivalent vaccine may be an effective strategy to reduce the burden and mortality of invasive NTS.

Mali is a land-locked country in West Africa that the United Nations ranks among the 10 least-developed countries [1], with the eighth highest under-5 childhood mortality rate in the world [2]. In 2002, a retrospective review of medical records of children admitted to l’Hôpital Gabriel Touré (HGT), the principal pediatric hospital in Bamako, revealed that most admissions were due to suspected infections, and 20% of admitted children in whom a serious infection was clinically suspected died in the hospital [3]. To characterize the infections that were responsible for these deaths, a clinical microbiology laboratory was established at HGT with blood culture capability to identify the etiology of these illnesses and determine the proportion that were due to vaccine-preventable infections.

Prospective hospital-based surveillance for bacterial infections began in 2002 and is ongoing [4]. The results have provided the impetus to introduce vaccines for infections such as *Streptococcus pneumoniae* and *Haemophilus influenzae* type b (Hib) into the routine vaccination schedule for infants under the Expanded Program on Immunization. Continued surveillance following introduction documented a marked reduction in the incidence and mortality related to these infections [5, 6]. This surveillance system also uncovered a substantial burden of invasive *Salmonella enterica* bloodstream infections in Malian children [7].

Invasive *S. enterica* infections are classified as either enteric (typhoid or paratyphoid) fever, caused by serovars Typhi, Paratyphi A, Paratyphi B, and rarely Paratyphi C, or nontyphoidal *Salmonella* (NTS), consisting of all other serovars. Population-based surveillance at 13 sentinel sites in 10 sub-Saharan African countries between 2010 and 2014 as part of the Typhoid Fever Surveillance in Africa Program (TSAP) found *S.* Typhi to be the most common pathogenic bacteria isolated (24%); the adjusted incidence rate per 100 000 person-years of observation ranged from 0 cases in Sudan to 383 at a site in Burkina Faso (median, 58) [8]. The peak incidence always occurred after infancy but varied by site. In recent decades, invasive NTS (iNTS) has emerged as a major cause of invasive bacterial febrile illness in many regions of sub-Saharan Africa [9], with several countries reporting a high incidence [10–14]. In the TSAP, 17% of pathogenic bacteria were attributable to NTS; the adjusted incidence rate per 100 000 person-years ranged from 0 to 237 cases (median, 19) across all age groups, but ranged from 0 to 1733 cases (median, 195.5) among infants, the highest-risk group [8]. Invasive NTS infections also exhibit considerable regional diversity, as well as a fluctuating burden over time, and a predilection for children under 5 years of age with human immunodeficiency virus (HIV) infection, malaria, and malnutrition [9].

To supplement these data, we describe 17 years of surveillance for pathogens causing invasive bacterial infections at HGT, particularly the contribution of *S. enterica*. We previously reported iNTS morbidity and mortality among children hospitalized at HGT from 2002–2014 [15]. Here, we expand our findings to examine temporal trends among hospitalized children from 2002 to 2018, describe the burden of iNTS in outpatients, examine the relationship between invasive bacterial infections and malaria, and report the burden of enteric fever in inpatients and outpatients.

## METHODS

### Study Enrollment and Procedures

Since obtaining blood cultures is not standard of care at HGT, it was offered as part of a research protocol. The method of screening and enrolling eligible children for the study has been previously described [15]. In brief, all children aged 15 years or younger seeking care at HGT were screened for inclusion into the study. Eligible patients had a high fever (axillary temperature of at least 39^o^C) or a clinically suspected invasive bacterial infection, including meningitis, pneumonia, empyema, peritonitis, pyomyositis, septic arthritis, osteomyelitis, and typhoid fever. Children were triaged for treatment either at the HGT inpatient unit (Inpatient Surveillance Cohort [ISC]) or treated in the outpatient setting and discharged home (Outpatient Surveillance Cohort [OSC]). Inpatient surveillance began on 1 June 2002 and is ongoing; results reported here include data collected up to 31 December 2018. Outpatient surveillance took place between 25 May 2005 and 31 October 2017 and was restricted to children aged less than 36 months.

Study personnel collected demographic and clinical information at presentation from all participants, including age, sex, place of residence, axillary temperature, clinical history, physical findings, laboratory and imaging study results (if available), and initial diagnosis, which they recorded onto standardized forms. For both inpatients and outpatients, they documented the child’s hospital course and clinical management until discharge or in-hospital death. When a putative pathogen was recovered from a sample obtained from an outpatient, study personnel attempted to complete a follow-up home visit to ascertain the child’s clinical outcome within 30 days of leaving the hospital. Culture results from inpatients and outpatients were reported to physicians overseeing the patient’s care.

A case of invasive *Salmonella* disease was defined as a patient with symptoms consistent with an invasive bacterial infection, from which culturing of a blood or other bodily fluid sample grew *S*. Typhi or *S.* Paratyphi A, B, or C for enteric fever, or an NTS serovar for iNTS.

### Specimen Collection and Microbiological Methods

The study physician drew blood for culture using aseptic technique (1–5 mL for infants, 3–5 mL for children aged 12–59 months, and 5 mL for children aged 5–15 years). Additional specimens for culture from normally sterile sites were obtained at the discretion of the treating physician and processed according to standard laboratory methods [16]. Samples were inoculated into BACTEC Culture Vials Peds Plus/F flasks (Becton Dickinson, Sparks, MD) and maintained at room temperature until transport to the bacteriology laboratory at HGT within 6 hours of collection. Then, samples were incubated for up to 5 days in an automated blood culture system (BACTEC 9050 from 2002 to 2015, BACTEC FX 40 from 2015 to present; Becton Dickinson). Bacteria were identified by Gram stain, culture, and biochemical testing using standard methods [16], and the results were reported to the clinician. Contaminants were defined as all gram-positive rods and coagulase-negative *Staphylococci* and were not considered putative pathogens. A thick blood smear for malaria parasites was also examined if possible.

Isolates identified as *S. enterica* were serogrouped and serotyped by the multiplex polymerase chain reaction method of Tennant et al [7] and Levy et al [17], frozen in trypticase soy broth, and shipped to the Molecular Diagnostic and Microbiology Section of the Center for Vaccine Development and Global Health at the University of Maryland School of Medicine for long-term storage. Testing by agglutination with antisera was performed as needed, as previously described [17].

### Data Management and Statistical Methods

During the 17-year study period, data were recorded on paper forms and then entered into EpiInfo (Centers for Disease Control and Prevention, Atlanta, GA) or Teleform (OpenText, Waterloo, Canada), except from 1 October 2007 to 11 September 2012 when data were directly entered in the Global Digital Health data-management system (Tel Aviv, Israel). Yearly frequency distribution of *Salmonella* serovars, as well as case fatality ratios, were calculated. Pathogen-specific annual incidence rates per 100 000 children in Bamako (with 95% confidence intervals [CIs]) were calculated by dividing the number of cases of each pathogen by census-derived, age-stratified population estimates collected by the National Institute of Statistics in Mali (INSTAT). Age-stratified yearly Bamako population estimates were extrapolated using a growth model with census-derived data points collected by INSTAT in 1999 and 2009; only Bamako residents were included in incidence calculations. Chi-square test was used for comparison of proportions, and statistical significance was evaluated at the .05 level (2-sided). Logistic regression was used to calculate age-adjusted odds ratios (ORs). Data analysis was generated using SAS software (version 9.4; SAS Institute, Cary, NC).

The protocol was approved by the Institutional Review Board at the University of Maryland, Baltimore, and the Ethics Committee of the Faculté de Medécine de Pharmacie et d’Odontostomatologie, University of Mali.

## RESULTS

### Participants

**Figure 1.**
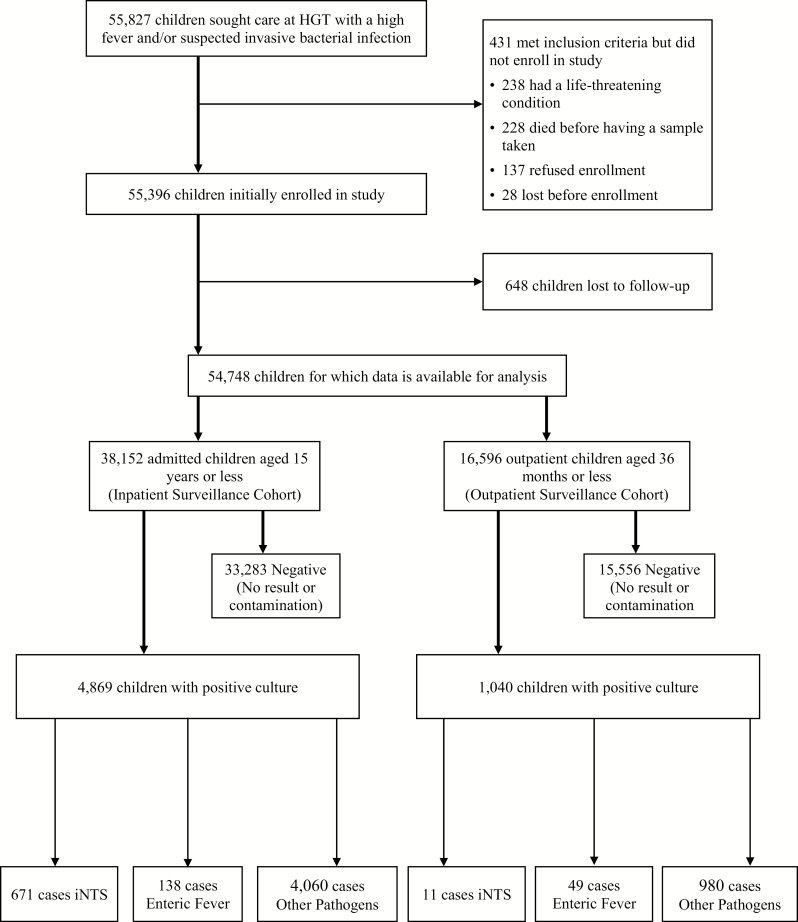
Study flow of participants. Abbreviations: HGT, Hôpital Gabriel Touré; iNTS, invasive nontyphoidal *Salmonella.*

A total of 55 827 children met initial criteria for enrollment in either the inpatient (ISC) or outpatient (OSC) study cohort (Figure 1); 431 were excluded and 648 were lost to follow-up after enrollment, making data available for 54 748 children.

Between 1 June 2002 and 31 December 2018, we enrolled and analyzed 38 152 children aged 15 years or younger in the ISC. Of these children, 4869 had a blood or bodily fluid sample taken that grew a putative pathogen (12.8%). There were 671 cases of iNTS (1.8% of enrolled inpatients, 13.8% of enrolled inpatients with a pathogen identified) and 138 cases of enteric fever (0.4% of enrolled inpatients, 2.8% of enrolled inpatients with a putative pathogen).

Between 15 May 2005 and 31 October 2017, 16 596 children aged 35 months or younger were enrolled and analyzed in the OSC. Of these children, 1040 had a blood or bodily fluid sample taken that grew a putative pathogen (6.3%). There were 11 cases of iNTS (0.07% of enrolled outpatients, 1.1% of enrolled outpatients with a putative pathogen), and 49 cases of enteric fever (0.3% of enrolled outpatients, 4.7% of enrolled outpatients with a putative pathogen). Over the 17 years of surveillance among all children analyzed in both cohorts, NTS was the third most prevalent pathogen isolated, behind *S. pneumoniae* and Hib; *S*. Typhi and *S*. Paratyphi C together ranked seventh.

### Descriptive Data

Characteristics of study participants for both cohorts are given in Table 1. In the ISC, the prevalence of iNTS was higher than the prevalence of enteric fever (1.8% vs 0.4%; *P* < .0001). For the OSC, the opposite was true; the prevalence of iNTS was lower than the prevalence of enteric fever (0.07% vs 0.3%; *P* < .0001). Among inpatients, the median age of iNTS cases was lower than the median age of enteric fever cases (1.8 years vs 5 years; *P* < .0001). Invasive NTS and enteric fever were significantly more prevalent in males than females in both the ISC (58.4% males; *P* < .0001) and OSC (90.9% male; *P* = .0001). In the ISC, iNTS was isolated from the following sites: 606 in blood alone, 55 in cerebrospinal fluid (with or without blood), 7 in subcutaneous fluid, 1 in pleural fluid, 1 in articular fluid, and 1 in peritoneal fluid. All 11 outpatient cases of iNTS were isolated from blood alone.

**Table 1.** Characteristics of the Inpatient and Outpatient Surveillance Cohorts

	No. of Cases	Males, no. (%)	Median Age in Years (SD)	Bamako Residents, no. (%)	No. of Malaria Smears Performed (%)	No. Malaria Positive by Smear (%)^a^	Case Fatality, no. (%)
Inpatient Surveillance Cohort							
iNTS	671	392 (58.4)	1.8 (3)	462 (68.9)	613 (91.4)	117 (19)	140 (20.9)
Enteritidis	258	145 (56.2)	1.8 (2.7)	247 (58.1)	247 (95.7)	47 (19)	66 (25.6)
Typhimurium	213	120 (56.3)	2 (3.3)	163 (76.5)	189 (88.7)	33 (17.5)	33 (15.5)
Dublin	79	53 (67.1)	2 (2.6)	56 (70.9)	76 (96.2)	17 (22.4)	12 (15.2)
I:4,[5],12:i:-	36	25 (69.4)	2 (4.5)	30 (83.3)	28 (77.8)	9 (32.1)	5 (13.9)
Other serovar	85	49 (57.7)	1.1 (2.6)	63 (74.1)	73 (85.9)	11 (15)	24 (28.2)
Enteric fever	138	85 (61.6)	5 (3.8)	108 (78.3)	123 (89.1)	18 (14.6)	11 (8.0)
Typhi	129	79 (61.2)	6 (3.8)	100 (77.5)	114 (88.4)	14 (12.2)	11 (8.5)
Paratyphi C	9	6 (66.7)	3 (1.4)	8 (88.9)	9 (100)	4 (44.4)	0 (0)
Outpatient Surveillance Cohort							
iNTS^b^	11	10 (90.9)	0.9 (0.7)	9 (81.8)	11 (100)	0 (0)	0 (0)
Enteric Fever	49	30 (61.2)	1.9 (0.6)	43 (87.8)	46 (93.9)	2 (4.1)	0 (0)
Typhi	44	28 (63.6)	2 (0.6)	38 (86.4)	42 (95.5)	2 (4.6)	0 (0)
Paratyphi C	5	2 (40)	1.5 (0.5)	5 (100)	4 (80)	0 (0)	0 (0)

Abbreviation: iNTS, invasive nontyphoidal *Salmonella.*

^a^Percentages of malaria positives are given as the proportion of positive malaria smears among patients who had a malaria smear.

^b^Eleven iNTS cases in the Outpatient Surveillance Cohort are composed of the following serovars: 6 Enteritidis, 1 Typhimurium, 2 Dublin, 1 Group D, and 1 Virchow.

Among iNTS cases in the ISC who had a malaria smear performed, 19% were positive. This proportion was similar among inpatients with enteric fever (14.6%; *P* = .25). The malaria smear positivity proportion was higher among inpatients with a negative blood culture (24.3%; *P* = .002) than that for iNTS cases. Compared with those with a blood culture positive for another putative pathogen, children with iNTS were twice as likely to have a positive malaria smear (age-adjusted OR, 2.0; 95% CI, 1.6–2.6). Similar trends were observed when comparing iNTS malaria smear positivity with those with a putative gram-positive blood culture (age-adjusted OR, 2.1; 95% CI, 1.6–2.8) and with those with a putative gram-negative blood culture other than iNTS (age-adjusted OR, 1.8; 95% CI, 1.4–2.3). Positive malaria smears were less prevalent among the OSC; none of the iNTS outpatients and 4% of the enteric fever outpatients tested positive.

### Serovar Distribution

**Figure 2.**
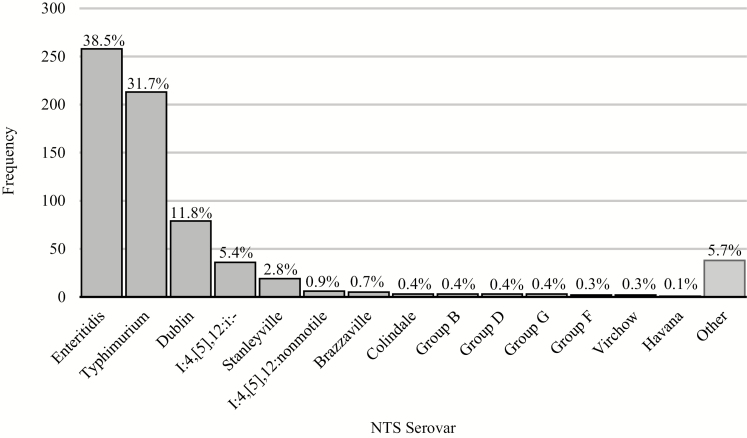
Serovar distribution of the 671 NTS isolates obtained via culture in the Inpatient Surveillance Cohort. Proportions of all NTS isolates attributable to each serovar are given. Abbreviation: NTS, nontyphoidal *Salmonella.*

The distribution of all NTS serovars among the ISC is displayed in Figure 2. *Salmonella* Enteritidis was the most common serovar, accounting for 38.5% of all isolates (n = 258), followed by *S.* Typhimurium (31.7%; n = 213), *S.* Dublin (11.8%; n = 79), and I:4,[5],12:i:- (5.4%; n = 36). These 4 predominant serovars accounted for 87.4% of all NTS isolated. The 11 NTS serovars isolated among the OSC included 6 *S.* Enteritidis isolates, 2 *S.* Dublin isolates, 1 *S.* Typhimurium isolate, 1 Group D isolate, and 1 *S.* Virchow isolate. Among residents of Bamako, *S.* Typhimurium was the most prevalent serovar isolated, comprising 34.8% of all iNTS. However, among nonresidents, *S.* Enteritidis comprised 50.2% of all iNTS.

Among inpatients, the 138 cases of enteric fever consisted of 129 *S.* Typhi isolates and 9 *S.* Paratyphi C isolates. For outpatients, the 49 cases of enteric fever comprised 44 *S.* Typhi isolates and 5 *S.* Paratyphi C isolates. Serovars *S.* Paratyphi A and *S.* Paratyphi B were not identified at HGT.

### Secular Trends

**Figure 3.**
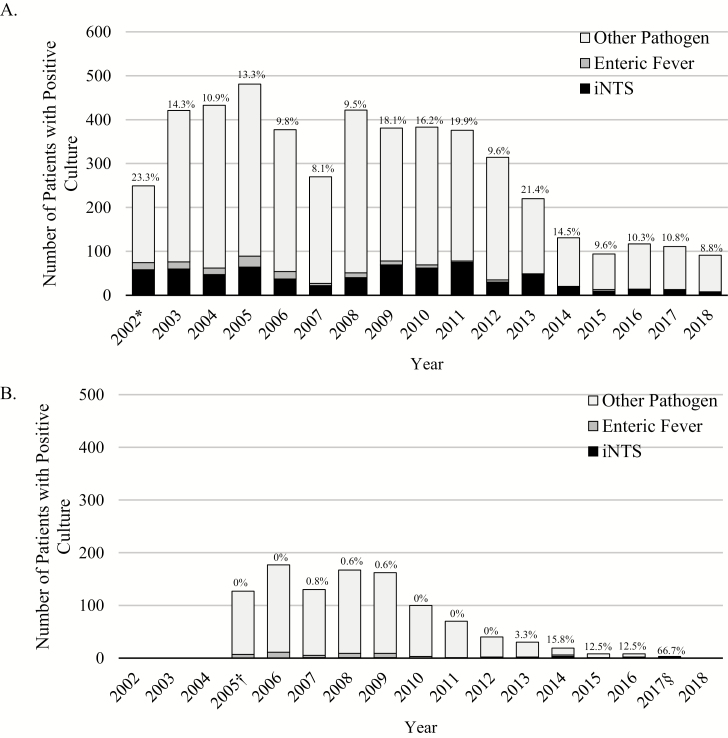
Distribution of iNTS, enteric fever, and other pathogens isolated each year for the ISC (*A*) and OSC (*B*). Proportions of all cultures attributable to iNTS are given. *2002 is an incomplete year of data collection for the ISC, as surveillance for bacterial pathogens began on 1 June 2002. ^†^2005 is an incomplete year of data collection for the OSC, as outpatient surveillance began on 15 May 2005. ^§^2017 is an incomplete year of data collection for the OSC, as outpatient surveillance ended on 31 October 2017. Abbreviations: iNTS, invasive nontyphoidal *Salmonella*; ISC, Inpatient Surveillance Cohort; OSC, Outpatient Surveillance Cohort.

The annual frequency of positive cultures, subdivided by predominant isolated pathogens and ISC and OSC, is shown in Figure 3. In the ISC, the frequency of positive cultures has decreased over time. Nevertheless, the proportion of positive cultures attributable to iNTS remains stable in the ISC; and between 2016 and 2018, 10% of all positive cultures were attributable to iNTS. In 2017, it was the third most frequently isolated pathogen, and in 2018 it was the fourth most common pathogen, behind *Staphylococcus aureus*, *S. pneumoniae*, and Hib. Enteric fever was relatively uncommon in recent years; between 2009 and 2018, there were 34 cases of *S.* Typhi and no cases of *S.* Paratyphi C among inpatients. The number of cultures analyzed for both the ISC and OSC has decreased in recent years (Supplementary Figure 1). The number of inpatients and outpatients screened at HGT, as well as the proportion who were enrolled, has also decreased (Supplementary Figure 2).

The frequency of cultures positive for any putative pathogen in the OSC was relatively high between 2005 and 2009, then began declining until surveillance was concluded in October 2017. Invasive NTS prevalence remained very low among outpatients throughout the study. Overall, *S.* Typhi and *S*. Paratyphi C accounted for 5% of all positive cultures, but frequency decreased over time such that only 1 case (*S*. Typhi) has been identified among outpatients since 2015.

**Figure 4.**
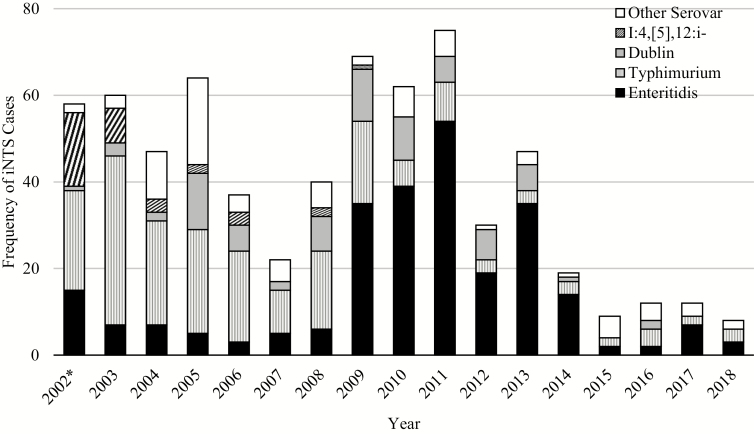
Distribution of predominant NTS serovars Typhimurium, Enteritidis, Dublin, I:4,[5],12:i:-, and Other Serovar each year among inpatients. *2002 is an incomplete year of data collection, as surveillance for bacterial pathogens began on 1 June 2002. Abbreviations: iNTS, invasive nontyphoidal *Salmonella*; NTS, nontyphoidal *Salmonella.*

**Figure 5.**
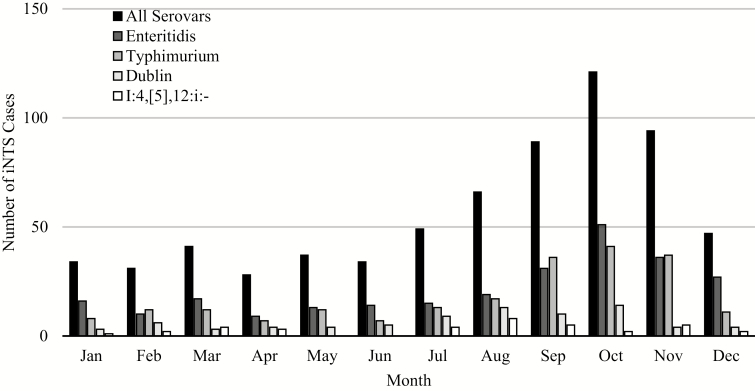
Seasonality of iNTS cases among inpatients: All Serovars, Typhimurium, Enteritidis, Dublin, and I:4,[5],12:i:-. Abbreviation: iNTS, invasive nontyphoidal *Salmonella.*

Between 2009 and 2018, *S.* Enteritidis was the most commonly encountered iNTS serovar, while *S.* Typhimurium, previously the more prevalent serovar, decreased in frequency (Figure 4). There have only been 3 *S.* Dublin cases since 2013, and no I:4,[5],12:i- serovars isolated since 2009. Additionally, iNTS appeared most frequently from August to November, towards the middle and end of the rainy season in Mali, peaking in October (Figure 5). This trend was observed for *S.* Enteritidis and *S.* Typhimurium.

### Mortality

For the ISC, case fatality among iNTS cases (20.9%) was significantly higher than for enteric fever (8.0%; *P* = .0004). Among NTS serovars, case fatality was significantly higher for *S.* Enteritidis (25.6%) compared with *S.* Typhimurium (15.5%) (*P* = .01). There was no difference in mortality between iNTS cases with a positive malaria smear and iNTS cases with a negative malaria smear (*P* = .62). For the OSC, there was no case fatality for iNTS or enteric fever; all 11 cases of iNTS and 49 cases of enteric fever were followed up with a home visit, and no death was recorded.

**Figure 6.**
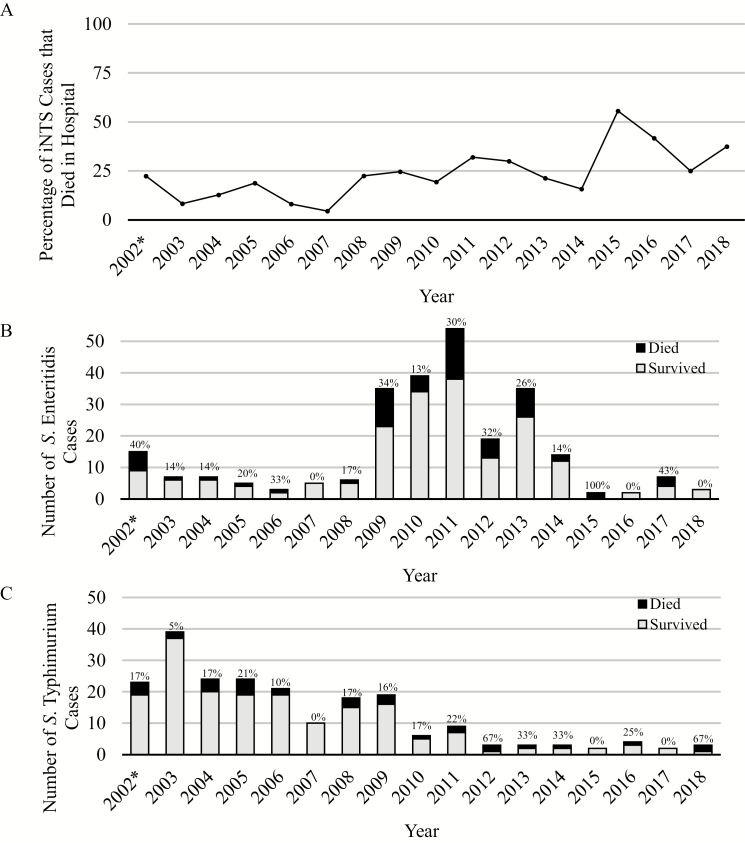
Annual case fatality ratios for all inpatients with a positive culture for NTS (*A*). Number of cases who survived (gray) and died (black) while hospitalized (*B*, Enteritidis; *C*, Typhimurium). Case fatality ratios each year are shown above each year for *S.* Enteritidis (*B*) and *S.* Typhimurium (*C*). *2002 is an incomplete year of data collection, as surveillance for bacterial pathogens began on 1 June 2002. Abbreviation: NTS, nontyphoidal *Salmonella.*

Invasive NTS case fatality remained high in recent years (39% between 2015 and 2018, inclusive), albeit with considerable year-to-year variation when the number of cases was small (Figure 6). Although these small sample sizes did not support statistical comparisons, some trends are notable: *S.* Enteritidis had a period of particularly high prevalence and mortality between 2009 and 2014, while *S.* Typhimurium had a higher prevalence between 2002 and 2009, with a lower overall mortality.

Some trends are apparent when case fatality ratios are stratified by age for all inpatient iNTS, *S.* Enteritidis, *S.* Typhimurium, and enteric fever cases (Table 2). In-hospital mortality for iNTS was inversely correlated with age. *Salmonella* Enteritidis case fatality was particularly high among infants aged less than 1 year (37.1%) as well as children aged 12–59 months (23.2%). Enteric fever produced a high case fatality ratio in infants (33%) but with a low sample size (9 cases); mortality for enteric fever was lowest among school-aged children (3.5%).

**Table 2.** Case Fatality Ratios of Nontyphoidal *Salmonella* Serovars and Enteric Fever Among the Inpatient Surveillance Cohort, Stratified by Age

	All iNTS Serovars				*S*. Enteritidis				*S*. Typhimurium				Enteric Fever			
Age Group	Cases, no.	Deaths, no.	CFR, %	95% CI	Cases, no.	Deaths, no.	CFR, %	95% CI	Cases, no.	Deaths, no.	CFR, %	95% CI	Cases, no.	Deaths, no.	CFR, %	95% CI
0–11 months	180	51	28.3	21.9–35.5	62	23	37.1	25.2–50.3	49	10	20.4	10.2–34.3	9	3	33.3	7.5–70.1
12–59 months	367	70	19.1	15.2–23.5	151	35	23.2	16.7–30.7	119	18	15.1	9.2–22.8	45	5	11.1	3.7–24.1
5–15 years	124	19	15.3	9.5–22.9	45	8	17.7	8. –32.1	45	5	11.1	3.7–24.1	85	3	3.5	.7–10
All ages	671	140	20.9	17.8–24.1	258	66	25.6	20.4–31.4	213	33	15.5	10.9–21.1	139	11	7.9	4–13.7

Abbreviations: CFR, case fatality rate; CI, confidence interval; iNTS, invasive nontyphoidal *Salmonella.*

### Incidence

**Figure 7.**
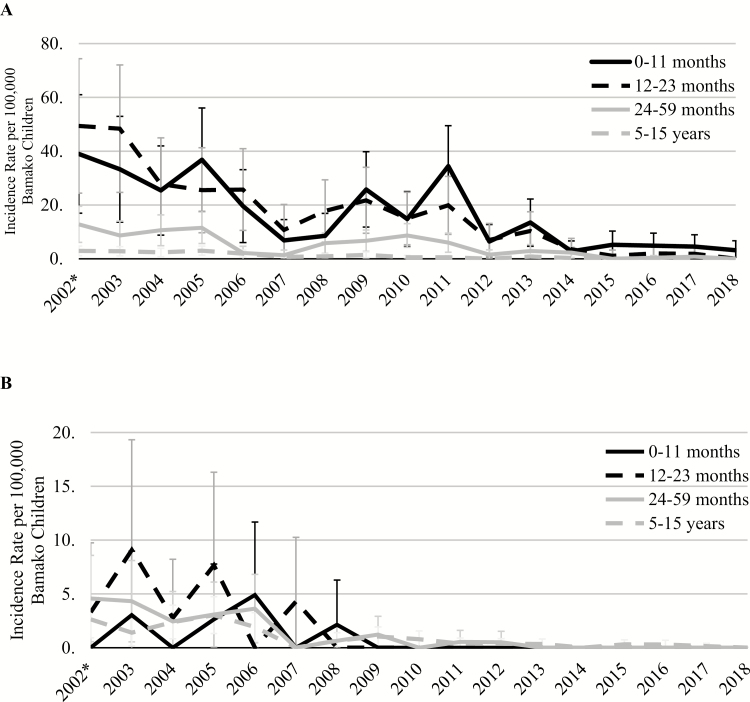
Age-stratified incidence rates per 100 000 Bamako children with 95% confidence intervals for iNTS (*A*) and enteric fever (*B*). *2002 is an incomplete year of data collection, as surveillance for bacterial pathogens began on 1 June 2002. Abbreviation: iNTS, invasive nontyphoidal *Salmonella.*

Overall iNTS incidence in Bamako decreased from 2002 to 2018 (Figure 7). Incidence was highest among infants aged 0–11 months and children aged 12–23 months, followed by children aged 24–59 months and children aged 5–15 years. Incidence for iNTS was higher than for enteric fever for every year, which has had a low incidence at every age group throughout the duration of surveillance.

## DISCUSSION

Although the incidence of iNTS has decreased in recent years, it continues to be a major pathogen. Nontyphoidal *Salmonella* was the third most commonly isolated bacterial pathogen found over the course of the surveillance study, behind *S. pneumoniae* and Hib, and disproportionately affects infants less than 12 months of age. Moreover, case fatality ratios remain unacceptably high in all age groups, ranging from 15.3% in 5- to 15-year-old children to 28.3% among infants.

*Salmonella* Typhimurium and *S.* Enteritidis are the 2 predominant NTS serovars encountered in this setting, and *S.* Enteritidis frequency has been increasing in recent years. Two observations about the epidemiology of iNTS emerge from our studies: one is the emergence of *S.* Enteritidis in 2009, overtaking *S.* Typhimurium as the predominant pathogen; second is the high case fatality of this emergent strain, exceeding *S.* Typhimurium in all age groups. Infants bear the greatest burden of *S*. Enteritidis infection; it was responsible for 34% of all iNTS infections among infants aged 0–11 months and had a 37% mortality rate. To our knowledge, similar trends have not been reported elsewhere; other studies in sub-Saharan Africa have reported *S*. Typhimurium as the predominant NTS serovar [8, 10–12, 18]. Ongoing surveillance elsewhere in sub-Saharan Africa would be of great interest to determine if this strain is disseminating to other locations.

Of note, iNTS had a low prevalence among outpatients, with only 11 cases found over 13 years. This may reflect the high severity of iNTS disease, indicating that it was more likely to be severe enough to require hospitalization at HGT. Enteric fever was relatively uncommon among children seeking care at HGT, comprising a mere 3 inpatient cases and 4 outpatient cases during the last several years of surveillance. Surveillance at HGT has been successful at identifying other major pathogens associated with invasive bacterial disease, including Hib, *S. pneumoniae*, and iNTS. The low prevalence and mortality from *S*. Typhi in Bamako suggest that it is not a pathogen of high public health importance in Bamako. Other studies have demonstrated the focal nature of enteric fever and iNTS, with high incidences in both urban and rural populations [8], so these data cannot be generalized to other regions in Mali.

Our findings support a large body of literature documenting an association between malaria and iNTS in sub-Saharan Africa [19] and a reduction in iNTS bacteremia that accompanies declining malaria prevalence. Some controversy remains, as some sites have shown an inverse relationship, which has been attributed, at least in part, to Berkson’s bias [20]. The association reported here (age-adjusted OR, 2.0) avoids Berkson’s bias by comparing malaria positivity between children with iNTS and children with non-iNTS bacteremia, as these 2 groups are expected to have comparable hospital attendance. The association between malaria and iNTS has been explained by a mechanism induced by *P*lasmodium *falciparum*, which impairs production of particular cytokines and hinders the function of macrophages and neutrophils, thereby increasing susceptibility to iNTS infection [21, 22]. Despite the higher risk for acquisition of iNTS, we did not find that iNTS cases testing positive for malaria had a higher fatality ratio than iNTS cases testing negative. In Malawi, the decline of iNTS incidence has been attributed to multiple public health interventions leading to reductions in malaria, HIV, and acute malnutrition [23]; a similar trend was reported in The Gambia [24]. In Mali, marked reductions in malaria incidence and moderate reductions in malnutrition in the face of persistently low HIV prevalence may have contributed to the declining incidence of iNTS that we have observed [25].

Several limitations of this study should be noted. Although we believe that the introduction of Hib and pneumococcal vaccines combined with reductions in malaria and malnutrition among infants and young children are likely to have caused reductions in the overall number of cultures positive for a putative pathogen, it is possible that changes in healthcare utilization for acute pediatric illness took place in Bamako over the 17 years of the study and contributed to the decrease in positive cultures accompanied by an increase in case fatality for iNTS at HGT. The number of patients screened at HGT has declined in recent years, and the proportion who met eligibility criteria and were enrolled decreased as well. This may indicate that healthcare utilization in Bamako has become decentralized. Based on data from HGT alone, the incidence of iNTS in Bamako children may have been underestimated. Age-specific healthcare-utilization behavior among Bamako communities is unknown but warrants additional investigation, as it may elucidate trends observed at HGT. The incidence of enteric fever based on cases identified at HGT may have underestimated the disease burden in Mali, since surveillance did not adequately address whether there may be pockets of enteric fever in more rural settings. Additionally, outpatient surveillance only took place among children aged less than 36 months. Because the median age for enteric fever among inpatients was 5 years of age, and mortality among typhoidal *Salmonella* cases was quite low, the study was not designed to capture milder, outpatient cases of enteric fever occurring among school-aged children. These factors likely resulted in an underestimation of the enteric fever incidence in Bamako.

Successful vaccination programs for pathogens such as *S. pneumoniae* and Hib have demonstrated that invasive pediatric diseases can be controlled [5, 6], and there is hope that development of a safe and effective vaccine for the control of iNTS disease could have similar benefits. There are 3 aspects of the epidemiology of iNTS disease in Bamako that would render a vaccination program useful. First, the burden of iNTS is mostly borne by infants and young children; 27% of all cases and 36% of all deaths were in children aged younger than 12 months, and 82% of all cases and 86% of all deaths were in children less than 5 years of age. Therefore, a vaccine that is incorporated into the Expanded Program on Immunization could reach those at greatest risk. Second, there is evidence that both iNTS and *S.* Typhi are spread person-to-person [26], suggesting that transmission could be interrupted with an effective vaccine. Third, there are 4 predominant serovars that contribute to 87% of all iNTS disease in this setting: *S.* Typhimurium, *S.* Enteritidis, *S.* Dublin, and I:4,[5],12:i:-. Thus, despite the large number of *Salmonella* serovars, a multivalent vaccine targeting a limited array of serovars could offer broad immunity against iNTS disease.

A Markov-chain transmission model was designed to evaluate the potential impact of a vaccine against iNTS if it were introduced into the Expanded Program of Immunization in Mali [27]. Bornstein et al [27] predicted that a multivalent efficacious vaccine with a level of immunization coverage similar to Hib conjugate vaccine would drastically reduce cases and deaths due to iNTS. Several bivalent *S.* Typhimurium and *S.* Enteritidis vaccines have been developed and are undergoing clinical trials [28–30]. The Center for Vaccine Development and Global Health, in partnership with Bharat Biotech International and the Wellcome Trust, has developed a trivalent *Salmonella* conjugate vaccine that includes a Vi conjugate (Typbar-TCV), in combination with *S*. Typhimurium and *S*. Enteritidis conjugates; this trivalent vaccine is currently in clinical trials [31]. These vaccines show promise to reduce the burden of iNTS disease in Mali and other countries in sub-Saharan Africa.

## Supplementary Data

Supplementary materials are available at *Clinical Infectious Diseases* online. Consisting of data provided by the authors to benefit the reader, the posted materials are not copyedited and are the sole responsibility of the authors, so questions or comments should be addressed to the corresponding author.

ciaa482_suppl_Supplementary_Figure_1

ciaa482_suppl_Supplementary_Figure_2

## Notes

***Author contributions.*** W. L. S. and K. L. K. drafted the manuscript with contributions from M. D. T. and M. M. L.. All authors reviewed the paper and made editorial suggestions. W. L. S. was responsible for final submission.

***Acknowledgments.*** The physicians and nurses of the Pediatric Service at HGT and in the clinical microbiology laboratory have been invaluable for their efforts. We also acknowledge Patrick Murray, James Campbell, the Malian Ministry of Health, the support staff at the Center for Vaccine Development, Mali, and the Malian communities.

***Financial support.*** This work was supported by the Bill & Melinda Gates Foundation, the Rockefeller Foundation, and the Optimus Foundation.

***Supp*** ***lement sponsorship.*** This supplement is funded with support from the Coalition against Typhoid Secretariat, housed at the Sabin Vaccine Institute in Washington, DC and made possible by a grant from the Bill & Melinda Gates Foundation.

***Potential conflicts of interest.*** S. M. T. is an inventor on US patent 9,050,283, “Broad spectrum vaccine against nontyphoidal *Salmonella*,” and US patent 9,011,87, “Broad spectrum vaccine against typhoidal and nontyphoidal *Salmonella* disease.” M. M. L. is co-inventor of a trivalent *Salmonella* (*S*. Enteritidis, *S*. Typhimurium, *S*. Typhi Vi) conjugate vaccine, a live oral *Salmonella* Typhi vaccine (CVD 909), and a live oral *Salmonella* Paratyphi A vaccine (CVD 1902). Bharat Biotech International (Hyderabad, India) has licensed the intellectual property to commercialize these vaccines. The trivalent *Salmonella* conjugate vaccine represents a partnership among the University of Maryland, Baltimore; Bharat Biotech International; and the Wellcome Trust, United Kingdom. M. M. L. reports the following issued patents: US patent 6,190,669, US patent 8,475,810, US patent 8,137,930, US patent 9,050,283, and US patent 9,011,871. M. M. L. also reports US provisional patent 62/146,545; International PCT/US2016/027325. All other authors report no potential conflicts. All authors have submitted the ICMJE Form for Disclosure of Potential Conflicts of Interest. Conflicts that the editors consider relevant to the content of the manuscript have been disclosed.
