# Supplementary figures and images for: Surveillance for Invasive Salmonella Disease in Bamako, Mali, From 2002 to 2018
Source: Clin Infect Dis. 2020 Jul 9;71(Suppl 2):S130–40. doi: 10.1093/cid/ciaa482 (PMC7388721; doi:10.1093/cid/ciaa482)

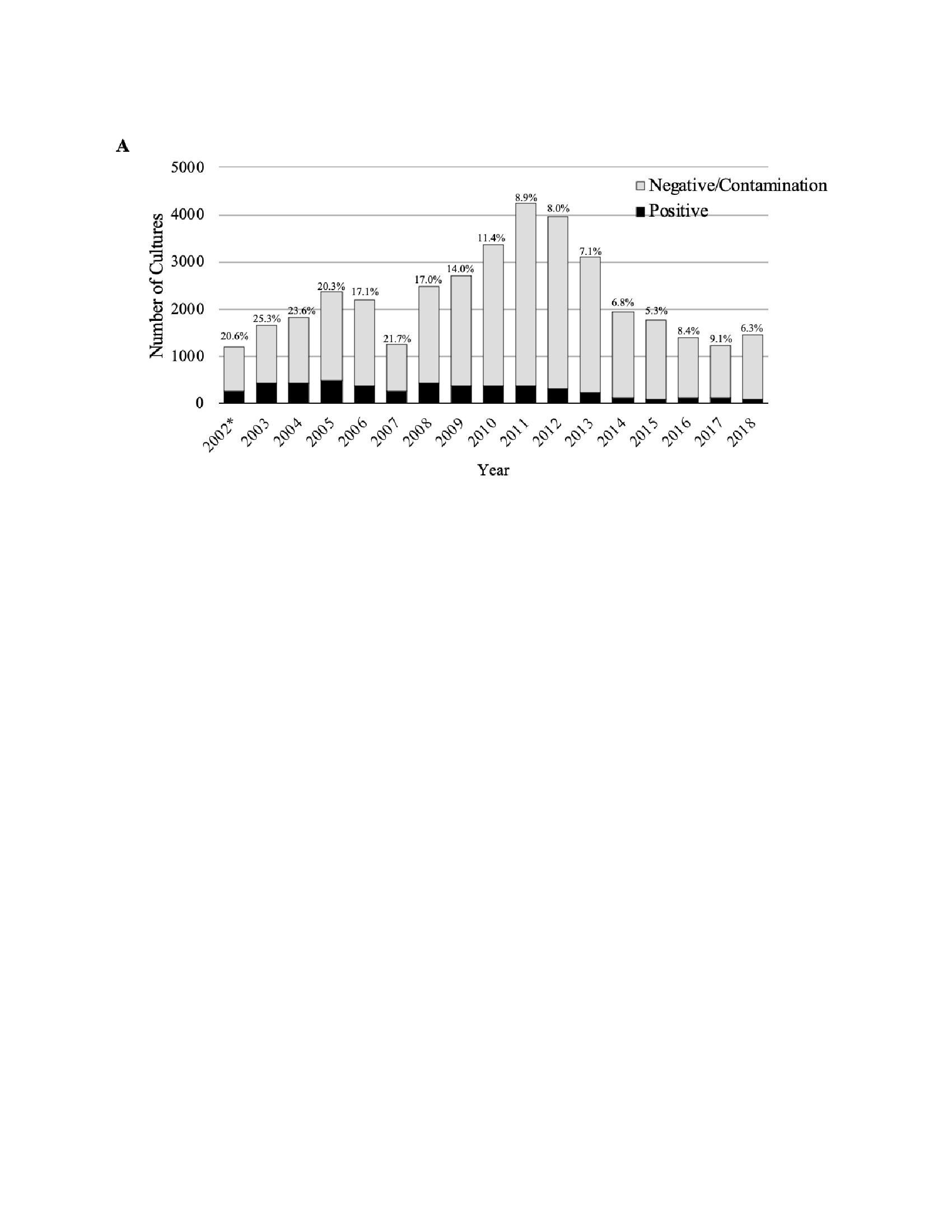

Supplement: ciaa482_suppl_Supplementary_Figure_1 [file ciaa482_suppl_supplementary_figure_1.png]

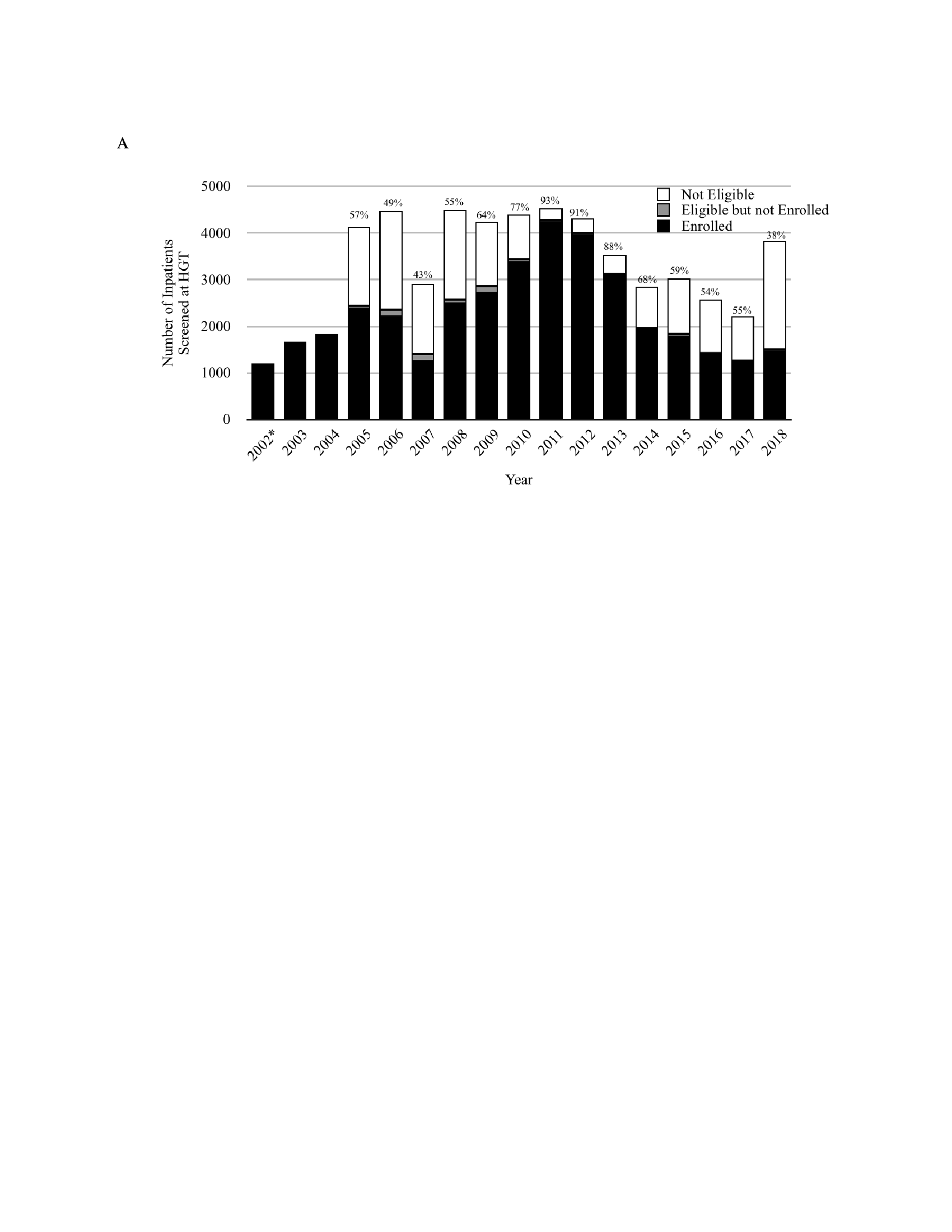

Supplement: ciaa482_suppl_Supplementary_Figure_2 [file ciaa482_suppl_supplementary_figure_2.png]
